# Effects of semantic relationship and preactivation on memory updating

**DOI:** 10.1007/s10339-022-01096-z

**Published:** 2022-05-13

**Authors:** Caterina Artuso, Francesco Bossi, Carmen Belacchi, Paola Palladino

**Affiliations:** 1 Department of Communication Sciences, Humanities and International Studies, University of Urbino, Urbino, Italy; 2 IMT School for Advanced Studies Lucca, Lucca, Italy; 3 University of Foggia, Foggia, Italy

**Keywords:** Working memory, Semantic memory, Semantic relationship, Memory preactivation

## Abstract

Semantic relationship modulates working memory (WM) processes by promoting recall but impairing recognition. Updating is a core mechanism of WM responsible for its stability and flexibility; it allows maintenance of relevant information while removing no-longer relevant one. To our knowledge, no studies specifically investigated how WM updating may benefit from the processing of semantically related material. In the current study, two experiments were run with this aim. In Experiment 1, we found an advantage for semantically related words (vs. unrelated) regardless of their association type (i.e., taxonomic or thematic). A second experiment was run boosting semantic association through preactivation. Findings replicated those of Experiment 1 suggesting that preactivation was effective and improved semantic superiority. In sum, we demonstrated that long-term semantic associations benefitted the updating process, or more generally, overall WM function. In addition, pre-activating semantic nodes of a given word appears likely a process supporting WM and updating; thus, this may be the mechanism favoring word process and memorization in a semantically related text.

## Introduction

### Long-term memory knowledge impact on working memory and updating

The two constructs of working memory (WM) and short-term memory (STM) refer to memories that are active over a brief period of time. However, while STM requires retention and subsequent recall of a given set of information (e.g., retention and recall of a new phone number), WM requires retention, and subsequent action, dependent on a given set of information (e.g., recognition that only 2 digits out of 7 are changed in the new phone number; thus the old, still relevant, 5 digits should be retained, while substituting the irrelevant 2 ones). The focus of the current work will be on WM and specifically how long-term knowledge impacts WM updating.

Thus, WM provides a framework for a series of interactive processes that include temporary storage and manipulation/processing of information, with an additional supervisory component and multiple storage components (Baddeley and Hitch 1974; Cowan 2017). One of the main issues raised with this classical model of WM concerns associations between verbal WM and long-term memory (LTM), an aspect that could potentially explain how semantic LTM influences the word sequence recall (Baddeley 1996). Baddeley (2000) included such a component (i.e., the episodic buffer), representing integration of information from different sources. Specifically, the buffer allows retrieval of information stored in LTM, and subsequently, its availability to WM for creation of episodic representations.

A link between WM and LTM components has been proposed via different WM models such as Cowan’s (1999), Conway and Engle’s (1994) and Oberauer and Hein’s ones (2012). Among the most well-acknowledged models of WM, the three-embedded component model (Oberauer 2002) represents an extension of the model proposed by Cowan (1999; see also Garavan 1998). The model distinguishes three WM components: the activated part of LTM (or activated LTM), the broad focus of attention and the single-item focus of attention. The activated LTM keeps potentially task-relevant information available, but some LTM-activated representations are held in the broad focus of attention. Contrary to activated LTM, which has potentially unlimited capacity, this broad focus of attention has limited capacity, is assumed to hold about four items (or chunks of information) available at a time, and to bind them into new structures (see also Schmiedek et al. 2009). Then, the narrower focus of attention (single-item) serves to select one item (or chunk) as the target of the next cognitive operation. Research showing evidence for the broad and narrow focus of attention has used a wide range of paradigms, but mainly digits as stimuli (such as arithmetic updating task; e.g., Oberauer and Hein 2012).

Studies on the role of LTM associations in WM have assessed the effects of enduring properties of verbal material (e.g., lexical and phonological) mainly via tasks involving recall (e.g., Hulme et al. 2003), recognition (e.g., Guerin and Miller 2008), or updating (e.g., Artuso and Palladino 2018). In general, it has been shown that lexical LTM associations facilitate the WM recall process (Hulme et al. 2003); specifically, the more strongly items are associated in LTM (e.g., more frequently associated), the more recall performance will benefit. On the other hand, studies on WM updating (see Artuso and Palladino 2018) demonstrated the opposite result: Strong sub-lexical associations from LTM are dismantled and updated with greater difficulty (i.e., they require longer RTs).

Updating information is one of the most crucial mechanisms through which WM works and may rapidly adapt to environmental change. It consists of selecting and maintaining available relevant information, and removing it away from memory once it is no-longer relevant; in other words, allowing modification of part of a representation in memory, while the rest remains unchanged [see seminal work by Morris and Jones (1990)]. Typically, achievement of an updating task is based on binding/unbinding processes between memory contents and/or actualization of bindings between contents (e.g., Artuso and Palladino 2011, 2014; Schmiedek et al. 2009).

The relationship between LTM and updating has been investigated rarely, and, to our knowledge, with reference to sub-lexical stimuli only (see Artuso and Palladino 2016, 2018, 2019). Here, the authors considered literature on the beneficial effects of highly associated LTM information based on lexical and phonological frequencies (see Gathercole et al. 1999; Hulme et al. 2003). Two initial studies investigated LTM sub-lexical associations updating between verbal materials (Artuso and Palladino 2016, 2018) in adult samples. There, participants engaged with the update of a three-item set, obtained by replacing a single-item whenever required. The task allowed collection of both online response times (RTs) during updating (i.e., dismantling of an item-set) and offline recognition accuracy/RTs after updating of a memory set to ensure updating effectiveness.

The strength of association between LTM stimuli was manipulated, in order to investigate modulation of the updating process itself. Overall, these studies clearly demonstrated that LTM associations modulate the updating process. In fact, these results suggested that strong associations are dismantled and updated with greater difficulty (i.e., they require longer RTs). A further study (with a developmental sample aged 7–10 years; Artuso and Palladino 2019) confirmed adult patterns; indeed, a substantial behavioral cost of dismantling and updating strong associations was shown, regardless of age.

In contrast, studies that focused on numerical material found facilitation effects during information updating (see Lendínez et al. 2014). When numbers involved in updating were more similar (i.e., in numerical distance), substitution occurred faster. Accordingly, the authors proposed that updating might be easier if the number presented is closer to the number stored in LTM. In other words, the greater overlap of feature sets, the greater item similarity, and the greater overall degree of overlap. Hence, when it is necessary to update a number sharing many features with another number stored in memory, the process can be performed more quickly; fewer features of the second number need activation, because shared features are already activated.

### Semantic LTM and short-term performance

Mandler et al. (1987) identified two different systems for organizing semantic memory (i.e., a type of declarative memory referring to general knowledge): taxonomic and thematic. Taxonomic organization is based on comparing traits across concepts; those that have many traits in common can be regarded as the same class of stimuli (e.g., class of fruit: apple, strawberry, pear). Thus, taxonomy refers to abstract categories including stimuli, hierarchically-organized, logically related to one another, linguistically coded and space/time independent. Conversely, thematic organization allows for objects of different taxonomic categories and with a low number of shared traits, to maintain a conceptual relation (e.g., apple, juice, pie). Thus, a thematic category refers to concrete stimuli, context-dependent and space–time situated.

Indeed, the first studies on semantic development (Lucariello et al. 1992; Nelson 1988) showed that children use thematic relations first (2–4 years), followed by taxonomic and hierarchical relations (i.e., logic/abstract categories; 7–8 years). This finding has been supported also by investigations into how semantic knowledge may modulate recall (e.g., Artuso et al. 2020; Belacchi et al. 2011; Belacchi and Palladino 2017; Giofrè et al. 2017; Monnier and Bonthoux 2011). Overall, these have shown that taxonomically related materials enhance recall preferentially (when compared to thematic ones).

In the same vein, most studies with adults showed the strong and direct interaction between LTM networks and recall performance, and the facilitating effects of semantic features on STM recall. For instance, Hulme et al. (2003) found that high-frequency words (vs. low) elicit better recall this was accounted for by recall of high-frequency words benefitting from stronger preexisting interword associations in the experimental context. To note, the effect is a lexical one, and not semantic. More related to semantics, Saint-Aubin et al. (2005) showed that semantic similarity is beneficial to *item* memory (due to the effects of an associative network and/or additional retrieval cues that support the recovery of items’ degraded representations). On the other hand, they found that semantic similarity is detrimental to *order* memory because it produces overlapping between representations (see also Poirier and Saint-Aubin 1996; Poirier et al. 2015; see also Majerus and D’Argembeau 2011, for an emotional/semantic account of the positive influence of LTM on STM recall).

On the other hand, a few studies showed the detrimental effect of semantic similarity.

Tse et al. (2011) investigated this topic in conjunction with serial recognition tasks: They designed trials in which words (semantically related or not) were presented sequentially; participants were instructed to remember the words and their presentation order. Immediately following presentation, they had to signal recognition of words, and initial study list order (i.e., same/different judgment). Participants’ responses were slower and less accurate for related lists than unrelated ones. Indeed, the authors showed that semantic relationship negatively impacts the ability to maintain serial order information. Notably, related lists were considered either associative/thematic (e.g., climb, mountain, peek) or categorical/taxonomic (e.g., aunt, cousin, dad), but no specific hypotheses or analyses were formulated on differential semantic association recognition.

Similarly, Guerin and Miller (2008) also reported that memory organization impaired recognition, when presenting lists of related vs. unrelated words. The authors accounted for a recognition impairment in an interesting argument. Indeed, words in related lists are more similar, but also less novel and distinctive. Other work indicates that when an item is unique, it is better remembered (e.g., Hunt 1995), and novel items are better remembered than familiar ones (e.g., Tulving and Kroll 1995). Therefore, in general, items in organized lists (regardless of similarity dimension) should not elicit high levels of item-specific memory. For this reason, recognition performance is poorer for related lists (i.e., with less distinctiveness) than for unrelated ones.

Recently, Ishiguro and Saito (2021) in a theoretical review using a meta-regression approach proposed to keep distinct semantic association vs similarity. They argue that the concepts of semantic association and similarity have been often confused, biasing studies. *Semantic association*, in the authors’ view, can be read as a ‘pre-experimental associative relationship between words and quantified via the associative strength values of free association norms (i.e., connectivity), which allows statistical control of the effect of semantic association on memory performance’ (Ishiguro and Saito 2021, page 388). On the other hand, *semantic similarity* can be manipulated and quantified via the strength of the manipulation, which is based on the three affective dimensions of valence, arousal and dominance. The idea that semantics can be represented in terms of a dimensional approach was taken (and adapted) from seminal studies by Osgood and Suci (1955) who devised the semantic differential method (see also Osgood 1952). Following this conceptualization, the authors found that semantic similarity is detrimental to order memory, while semantic association is beneficial to item memory. Of course, despite being an original and promising approach, it needs to be extended to tasks other than recall (i.e., a STM task) and to more complex conceptual tasks/manipulations, such as WM updating tasks, more resource demanding.

### The current study

The studies above considered showed a facilitating effect of LTM semantic knowledge on STM performance mainly, with a few others demonstrating detrimental effects. It is therefore worth noticing that all these studies used STM tasks (i.e., immediate serial recall or recognition). To our knowledge, there are no studies that specifically investigated how semantic LTM may impact WM performance (e.g., dual tasks) or WM updating. Therefore, we will formulate our hypotheses starting from the existing literature (i.e., STM one) taking into account the absence of specific literature on the interaction between semantic LTM and WM.

Updating is a core mechanism of WM responsible for its stability and flexibility and allows maintenance of relevant information while removing no-longer relevant one. In the current study two experiments were run to investigate the role of semantic relationship on WM updating, using an *n*-back task.

This task was firstly introduced by Kirchner (1958) and then largely used to assess WM (e.g., Jaeggi et al. 2010; Jonides et al. 1997). It requires participants to respond whenever the current stimulus matches one presented *n* positions back in the sequence (i.e. *n* depending on the load, i.e. 1, 2 or 3 filler words back). The novelty of the current study lies in the attempt to combine two research lines, sketched in the previous sections. That is, we are combining the role of LTM semantic knowledge on WM recognition via an *n*-back task, a task crucial to support discourse comprehension/production of semantically-related words. To our knowledge in fact, no studies have administered the *n*-back task with this aim, i.e., exploring how semantically related material is updated and how updating may be affected by semantic relationship between words.

A methodological digression is relevant here. The *n*-back is a task requiring a participant’s response whenever the current stimulus matches one presented *n* positions back in the sequence. It is intrinsically different from other updating tasks such as Morris and Jones’ (1990) running memory task, or those previously described (e.g., Artuso and Palladino 2011, 2018).

In fact, in Artuso and Palladino’s task (e.g., 2011, 2014) the participant had to unbind an association between items (i.e., removal of a single item from the memory set) and then to construct a new association (i.e., substitution of the previously removed item with a new one). This actively modifies a mental representation, via accommodation of a new input (Morris and Jones 1990). In addition, this substitution was shown to create a general cost for updating (i.e., longer response latencies; e.g., Artuso and Palladino 2011, 2018) and more specifically, a cost when stimuli are strongly bound in LTM (Artuso and Palladino 2016, 2018). Of note, in this updating task, only phonological associations between letters (i.e., low-level processing) were manipulated and not associations between words and their meaning (i.e., high-level semantic processing).

A task such as the *n*-back requires continuous monitoring of incoming information (e.g., words) and recognition of probed words, whenever the participant encounters a match between the current stimulus and the one presented *n* positions back in the sequence. In our view, this task comprises no active reorganization of memory representation (i.e., as requested by other tasks; see e.g., Artuso and Palladino 2018; Lendínez et al. 2014); instead, this mainly represents a recognition operation. Here, we decided to use the *n*-back task to examine how updating operates in the flow of information; a common situation in which we process continuous incoming information to connect and match this. An everyday example of such processing would be as in verbal communication, where we need to extract the most relevant/critical information among that presented.

In brief, Experiment 1 was designed to evaluate how semantic relationship impacts WM updating. Following previous studies, we created taxonomic and thematic semantic associations that have elicited differential memory performance. Therefore, we aimed to verify the performance impact of semantically related materials updating (versus unrelated) whether in terms of benefits (e.g., Belacchi et al. 2011; Saint-Aubin et al. 2005) or costs (e.g., Tse et al. 2011; see section "Experiment 1").

A second experiment was designed to boost semantic preactivation effects. We expected that encoding (and subsequent recognition) of a specific target word activates its related words and this preactivation of related words increases its accessibility and in turn enhances the encoding/recognition of the target word (see, e.g., Stuart and Hulme 2000) (see section "Experiment 2"). A pilot study was conducted to ensure the validity of the stimulus words.

#### Pilot study

A preliminary experiment was run to test the strength of the associative links between the three conditions we investigated (arbitrary, taxonomic and thematic relations). See Appendix 1 for full details on the experiment. In brief, findings demonstrated the equivalence of the strength of the associative links between the two conditions (i.e., taxonomic and thematic) and that both were more associated than the arbitrary conditions.

Further, to control for any bias in words selection, and be sure that results obtained are direct consequence of the type of semantic relation and not due to specific words chosen, we created two parallel sets of stimuli (set A original, set B control) where the same target word was part either of a taxonomic relation or a thematic relation. For example, the word *bed* in one set (A) was part of a taxonomic association (*furniture-chair-bed*), whereas in a parallel set (B) was part of a thematic association (*pillow-blanket-bed)* and so on. All these sets are reported in Appendix 2, both in original language (Italian) and in English.

In a recall task, modeled after the task devised by Belacchi and Palladino (2017), a total of 58 participants (17 males) took part in the pilot study. They were university students, recruited as volunteers to fulfill course credit, with no payment. The mean age was 23.24 years (SD = 2.26 years; age range 20–35 years). All participants provided their informed consent and were naïve to the purposes of the experiment. The study was conducted in accordance with the Ethical Standards laid down in the 1964 Declaration of Helsinki and the standard ethical procedures recommended by the Italian Psychological Association (AIP).

The sample was randomly divided into two groups, 29 participants each. The first group was administrated the set A, the second group set B, as between-participants variable. The analysis verified the absence of differences between set A and set B on recall accuracy, *F*(1, 56) = 0.11, *p* = 0.74. Therefore, the same target word was recalled similarly, either when belonging to a taxonomic association, or to a thematic one. We have thus demonstrated that our stimuli are balanced and results are unbiased from words selection. In the following experiments, therefore, we used the original set A.

#### Experiment 1

Experiment 1 was conducted to examine whether semantic relationship (i.e., taxonomic or thematic) modulates updating, compared to unrelated material. Considering the specific features of the task above, we formulated two opposing predictions, according to the evidence from different experimental perspectives.

As first prediction, we hypothesized that if during the task, the participant needs to dismantle a semantic association between words (as, for example, in Artuso and Palladino 2018), then we would expect a recognition cost, with longer RTs and diminished accuracy. Indeed, consistent with recognition studies (e.g., Guerin and Miller 2008; Tse et al. 2011), items in organized lists should not obtain high levels of item-specific memory. For this reason, recognition performance should be poorer for related lists (due to their lower distinctiveness), than for unrelated ones, and thus, semantic association would be detrimental.

The alternative hypothesis would predict that if during stimulus presentation, the participant does not need to dismantle (nor reorganize) any semantic association (i.e., given no explicit task demand to this effect, there is only simple stimulus exposure), then we expect a recognition benefit (e.g., Belacchi et al. 2011; Lendínez et al. 2014). In this instance, the participant should search in memory for the most activated word (i.e., a word from a semantic association should be recognized faster than without it), and the most recently seen word (i.e., 1-back trials) should be easier than 3-back trials. These effects should be observed both on accuracy and RTs.

In addition to these two constrasting predictions, and in line with previous findings (e.g., Belacchi et al. 2011; Belacchi and Palladino 2017), we also expected taxonomic relations to exert better memory support; however, it is worth noticing that these studies were conducted on developmental samples and used a recall procedure that differs from our current recognition paradigm.

On the contrary, in line with other findings in adult sample (Belacchi and Artuso 2018), we predicted the absence of differences between taxonomic and thematic associations and only a general semantic superiority effect over arbitrary associations (e.g., Giofrè et al. 2017).

## Methods

### Participants

A total of 25 participants (4 males) took part in the experiment. The sample size was estimated by performing a power analysis (see section “Statistical analyses”). They were university students, recruited as volunteers to fulfill course credit, with no payment. The mean age was 24.01 years (SD = 3.82 years; age range 21–26 years). All participants provided their informed consent and were naïve to the purposes of the experiment. The study was conducted in accordance with the Ethical Standards laid down in the 1964 Declaration of Helsinki and the standard ethical procedures recommended by the Italian Psychological Association (AIP).

### Stimuli

We used triplets of words taken from lists used in Belacchi and Palladino (2017) and Giofrè et al.’s (2017) studies. All words were of medium–high lexical frequency, taken from the Italian database (Marconi et al. 1994).

Words were grouped into triplets to form a taxonomic or thematic association; for the taxonomic one, the superordinate term was always presented in the first position, such as season-winter-summer. Triplets of words were interspersed with filler words (i.e., words with no association).

An example of a *3*-back triplet with a taxonomic association would be *season-winter-summer*. Then, two fillers are presented (*seat, hair*). The target word could be *summer*: with the word *summer* matching the stimulus two positions back in the sequence (i.e., 2 filler words). For each type of semantic association (i.e., taxonomic or thematic), we had 12 triplets of words; 36 taxonomic words and 36 thematic words, interspersed with 1, 2 or 3 filler words, for *1*-back, *2*-back or *3*-back, respectively.
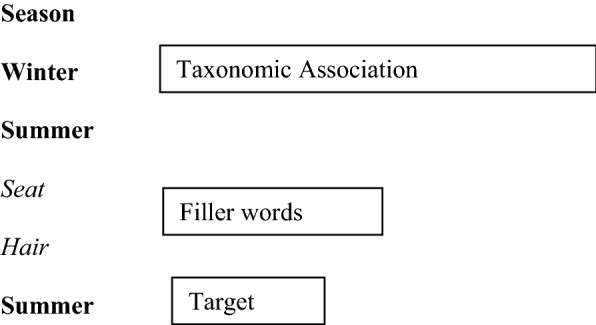


### Apparatus and procedure

Task administration was computerized for the *n*-back task, with the experiment run on a standard pc using the software Open Sesame (Mathôt et al. 2012). Each trial consisted of a stimulus-word written in white (in 70 Mono), presented in the center of the black screen for 1500 ms, and followed by the next stimulus. Here, the test comprised 3 levels of difficulty: *1*-back, *2*-back and *3*-back. Participants were instructed to respond whenever the current stimulus matched one presented *n* positions back in the sequence (i.e., *n* dependent on load; that is, 1, 2 or 3 filler words). The order of trials was randomized between participants. Participants were instructed to respond as quickly and accurately as possible by pressing the spacebar. Performance was assessed in terms of response time (RT) and accuracy, these serving as dependent variables. No feedback was given during the task. Three practice trials (one for each *n*-back condition) were administered before the experimental session started. The experimental session lasted about 15 min. See supplementary material for an example of the task, and an experimental output.

### Statistical analyses

To calculate the sample size for Experiment 1, an a-priori power analysis was performed by using the software G*Power (Faul et al. 2009). The effect size was fixed by taking into account the effect size of an experiment developed on the same paradigm (i.e., *n*-back) (Jaeggi et al. 2010). We considered the effect size the authors found on the load * task interaction effect on accuracy (i.e., *η*^2^_p_ = 0.37), since we wanted to estimate the load * semantic relationship effect size in our experiment. Given the absence of specific literature on the semantic relationship topic, this interaction effect was the most likely to be considered, in an identical task. In the power analysis, the effect size for repeated measures ANOVA (i.e., *f*(*U*)) was derived from the *η*^2^_p_ (*f*(*U*) = 0.766), *α* error was set to 0.05, the power (1 – *ß*) was set to 0.95, with 1 group of participants and 3 measurements each (for the 3 semantic relationships). The result suggested a sample size of 16 participants, but we decided to collect data from 25 participants to reach a higher power.

In Experiment 1, statistical analyses were focused on the analysis of target items.

Analyses on target items were carried out by using a generalized mixed effects model (on a binary distribution) on accuracy (coded as 0–1), and a linear mixed effects model on log-transformed response times (RTs). Log-transformation is considered best practice and one of the most used methods to deal with non-normally distributed data (as in the case of RTs; Ratcliff 1993; Cousineau and Chartier 2010). Both models included semantic relationship (arbitrary, thematic, taxonomic) and load (*1*-back, *2*-back, *3*-back) as independent fixed factors (in a full 3 × 3 factorial design) and random intercepts. We included only random effects that allowed models to converge (i.e., random intercepts). In the model for accuracy, *p* values were obtained by the models’ comparison method, i.e., comparison of deviation in two models (with the effect of interest vs. null model) in a chi-squared test. All parameters from the models are specified in tables in Appendix 3 section. In the model for RTs, only correct trials (accuracy = 1) were considered. Outliers exceeding 2 SDs from the mean RT were excluded, with mean and *SD* computed individually for each participant. Outliers removal was used in addition to log-transformation of data not only to increase normality in the distribution but also for a theoretical reason, i.e., remove data from trials in which participants lost their focus on the task. The significance of each effect in this model was estimated using the Satterthwaite approximation for degrees of freedom. *p* Values in all post hoc multiple comparisons were adjusted according to the Tukey HSD method.

## Results

### Models for target items

#### Accuracy

**Fig. 1:**
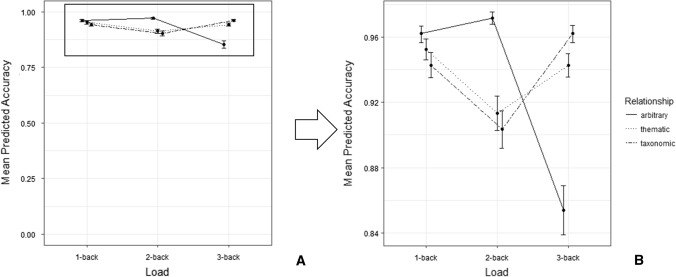
**A** represents the whole range of accuracy scores (from 0 to 1), while **B** shows a zoom on the subscale to show the actual accuracy

The model on participants’ accuracy showed a statistically significant interaction effect of semantic relationship * Load: *χ*^2^(4) = 14.382, *p* = 0.006. See Fig. 1.

Post hoc comparisons, based on semantic relationship, showed that participants were significantly more accurate in taxonomic (*M* = 0.962) than arbitrary (*M* = 0.854) items, in the *3*-back condition (*z* = -2.557, *p* = 0.028). The difference between thematic (*M* = 0.943) and arbitrary showed a statistical trend in the *3*-back condition (*z* = − 2.077, *p* = 0.095), however no significant differences emerged between taxonomic and thematic relationships (*z* = − 0.656, *p* = 0.789), or in any other load (all *z*s < 2, all *p*s > 0.1). Comparisons based on load showed significant differences in the arbitrary items only. Here, participants were more accurate in *1*-back (*M* = 0.962) and *2*-back (*M* = 0.972) vs. *3*-back (*M* = 0.854; *z*_1–3_ = 2.557, *p* = 0.029; *z*_2–3_ = 2.762, *p* = 0.016) trials. Items with taxonomic and thematic semantic relationships did not show significant differences based on load (all *z*s < 1.7, all *p*s > 0.2).[query section].

##### RTs

**Fig. 2:**
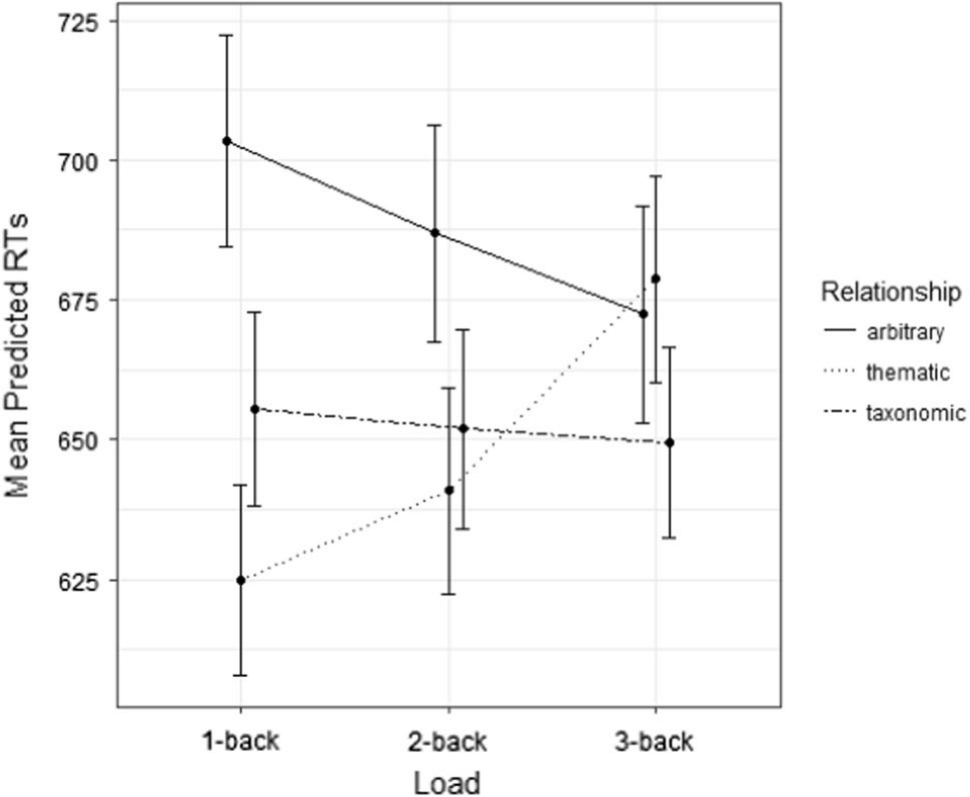
Experiment 1: mean predicted RTs (ms) as a function of semantic relationship and load. Dots represent mean values, and the error bars represent 95% confidence interval

The model for participants’ RTs showed a main effect of Semantic relationship (*F*(2, 764.23) = 10.354, *p* < 0.001) and Semantic relationship * Load interaction (*F*(4, 764.22) = 3.046, *p* = 0.017). See Fig. 2.

Multiple post-hoc comparisons were performed on the interaction, which further specified the main effect. The comparisons, based on semantic relationship, showed significant differences in the 1-back load, where participants were slower in arbitrary trials (*M* = 703 ms) than in thematic (*M* = 625 ms; *t*(764) = 4.891, *p* < 0.001) and taxonomic (*M* = 655 ms; *t*(764) = 2.860, *p* = 0.012) ones; thematic and taxonomic trials did not differ (*t*(764) = 2.013, *p* = 0.109). Comparisons based on load showed that, in thematic trials only, participants were significantly faster in *1*-back (*M* = 625 ms) than *3*-back Loads (*M* = 679 ms; *t*(764) =  − 3.218, *p* = 0.004). Again, for arbitrary and taxonomic trials, no significant differences emerged (all *t*s < 1.3, all *p*s > 0.3).

## Discussion

These results support a hypothesized semantic beneficial effect for recognition performance. Indeed, here, we found that words from semantic associations are recognized more accurately and faster than unrelated ones. In particular, for RTs, this pattern reached significance in the *1*-back condition. For accuracy, although a ceiling effect was evident, we found a semantic advantage for the *3*-back condition (i.e., the most demanding load condition), similarly to that observed by Belacchi et al. (2011).

These results contrast with findings of semantic relationship costs (e.g., Guerin and Miller 2008; Tse et al. 2011); we could hypothesize that these findings are related to the tasks used that are likely to involve more explicit processing of the words, contrary to the *n*-back. Indeed, for the *n*-back task, there is no explicit word meaning processing, i.e., the participant can even perform the task without knowing word meanings. In addition, the *n*-back did not involve demanding task components (e.g., presentation order recall), but recognition only.

It is worth highlighting that we did not find differences between taxonomic and thematic relationship, as both elicited better (and faster) recognition than unrelated words. However, the absence of a specific semantic organization effect is not unexpected. For instance, Belacchi and Artuso (2018) reported that taxonomic and thematic associated words are used flexibly in adulthood, with a slightly boosting effect of taxonomies. However, the task they used was highly resource demanding (i.e., dual task, with increasing attentional load requirements), while the *n*-back, as noted above, is a straightforward recognition task.

A limitation should be acknowledged. Accuracy reached ceiling effects, so the findings previously discussed should be interpreted with caution. On the other hand, accuracy, if considered as a control measure, gives important indication on the effectiveness of the task completion.^1^

### Experiment 2

A second experiment was designed to replicate Experiment 1 findings and to focus more effectively on how semantically related (or not) stimuli produce a behavioral advantage, compared to arbitrarily linked words (e.g., Giofrè et al. 2017). To this end, we manipulated the preactivation of the stimuli boosting their encoding (e.g., Stuart and Hulme 2000). The *preactivation monitoring framework* (e.g., Reyna and Lloyd 1997; Roediger et al. 2001) posits that the human lexicon organizes words based on their semantic properties, which implies that words with similar meanings are more strongly linked to each other compared to words with different meanings. The processing of one word can thus activate semantically related words via spread of activation*.*

Accordingly, following the preactivation framework, our predictions relate to stimuli features; we expect that preactivated words are encoded more deeply and are recognized more rapidly, compared to words that are not. Through the preactivation, we aimed to boost the semantic association advantage shown in Experiment 1, where semantic-related words are better recognized than unrelated ones. Given that we did not find differences between taxonomic and thematic relationship in Experiment 1 (both are better and faster recognized than unrelated words), we did not expect differences between taxonomic and thematic preactivation.

## Methods

### Participants

A total of 30 participants (5 males) took part in the experiment. The sample size was estimated by performing a power analysis (see section “Statistical analyses”). None of them had participated in Experiment 1. They were university students, recruited in order to fulfill course credit, and received no payment. The mean age was 23.74 years (SD = 4.21 years; age range 22–27 years). As in Experiment 1, all participants provided their informed consent and were naïve to the purposes. The study was conducted in accordance with the Ethical Standards laid down in the 1964 Declaration of Helsinki, and the standard ethical procedures recommended by the Italian Psychological Association (AIP).

### Task and procedure

The n-back task was identical to that administered in Experiment 1, as was the Procedure. In addition, before starting the experimental session, we administered a vocabulary test to assess participants’ linguistic skills. Moreover, a semantic preactivation task was implemented before running the n-back task. The experimental session lasted about 50 min.

### Linguistic assessment

#### Vocabulary

This test is taken from the Wechsler Assessment Intelligence Scale (WAIS) IV. Here, the participant is requested to define 30 words read aloud by the researcher. A mean percentage of 81% correct word definition was seen across the sample. This was used as covariate in all analyses. As the semantic n-back task is based on semantic word knowledge, this vocabulary test was used to control for general verbal knowledge across participants.

### Semantic preactivation phase

Semantic preactivation was produced with a semantic fluency task administered before the n-back task. The experimenter read a target word and asked the participant to say the first three words that came to their mind, either (1) belonging to the same category as the target word (taxonomic preactivation), or (2) associated with that target word, according to their belief (thematic preactivation). For example, if the experimenter said *medical doctor* (taxonomic preactivation), participants might name *cardiologist, pediatrician* and *orthopedic*. Alternatively, if the experimenter said *shoe* (thematic preactivation), participants might name, *lace, sole* and *sock.*

Once the experimenter ascertained the participant had understood instructions as well as the difference between taxonomic and thematic word association, the experimental session started. The full list of target words used to activate semantic knowledge is reported below. Half of the participants were given taxonomic and thematic targets from list i; the other half from list ii (in order to counterbalance preactivated items between participants).

Taxonomic targets

- List (i) Store, season, plant, bug, furniture, individual.
- List (ii) Music, color, animal, fruit, subject, dessert.

Thematic targets

- List (i) Light, bottle, yeast, cup, bench, desk.
- List (ii) Roof, purr, petal, sink, mane, blade.

### Statistical analyses

For Experiment 2, the sample size was estimated based on the effect we obtained in Experiment 1, as it was the best marker of the same effect in the second experiment. Indeed, an a-priori power analysis based on the specific effect size obtained in Experiment 1 is the best practice compared to an a-priori power analysis based on an approximation of the effect obtained from a similar paradigm (as we performed in Experiment 1 power analysis). The statistical power of the load * semantic relationship interaction effect on RTs in Experiment 1 was estimated by using a post hoc power analysis. Although there is still no complete agreement on the best practice to run a power analysis in a mixed effects model, the most reliable method to our knowledge is simulation-based power analysis (Brysbaert and Stevens 2018). By using this method with the “simr” package (Green and MacLeod 2016) in *R*, we obtained a power of 0.80 in Experiment 1. In order to obtain a power of 0.95, we approximated the effect size from Experiment 1 (*f*(*U*) = 0.318) and run a new a-priori analysis based on that effect size, α error = 0.05, power (1 – *ß*) = 0.95, with 1 group of participants and 9 measurements each (for the 3 semantic relationships * 3 cognitive load levels). Thus, we found a required sample size of 30 participants in order to obtain a statistical power = 0.95.

In Experiment 2, statistical analyses focused on semantic preactivation on target items. Analyses were carried out with the same methods used in Experiment 1. Vocabulary score (see Methods above) was used as covariate in all analyses. Analyses on effects of preactivation were performed only on target trials with thematic or taxonomic relationships. By definition, items with an arbitrary relationship did not have any relationship with preactivated words and, therefore, could not be influenced by semantic preactivation. Two models were performed on accuracy and RTs, identical to those used on target items, but with one additional independent fixed factor: preactivation (2 levels: item preactivation/no item preactivation for that participant).

## Results

### Models on preactivation effects

#### Accuracy

**Fig. 3:**
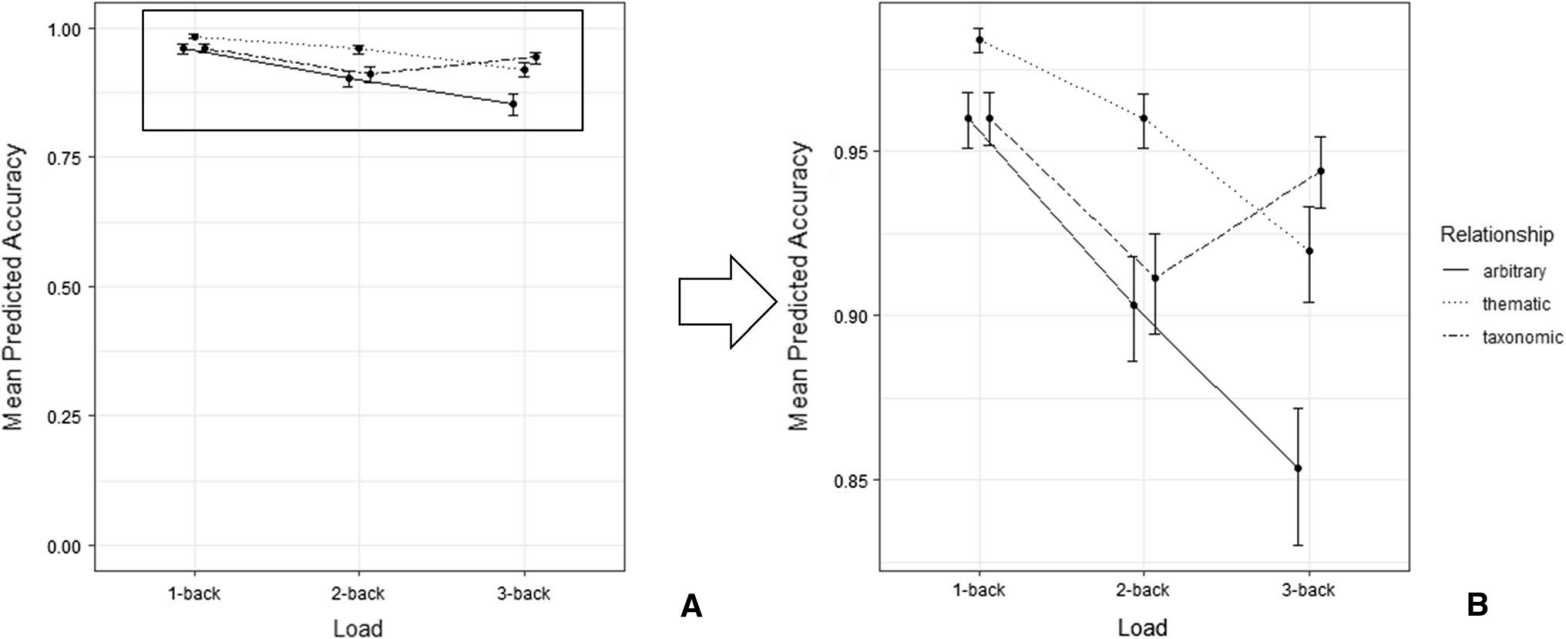
**A** represents the whole range of accuracy scores (from 0 to 1), while **B** shows a zoom on the subscale to show the actual accuracy

No effects of preactivation or interaction including preactivation emerged (all *F*s < 0.9, all *p*s > 0.38). As in Experiment 1, accuracy values showed a clear ceiling effect (*M* = 0.94, *SD* across participants = 0.09; see Fig. 3). For information concerning all specific parameters, see Appendix 3.

##### RTs

**Fig. 4:**
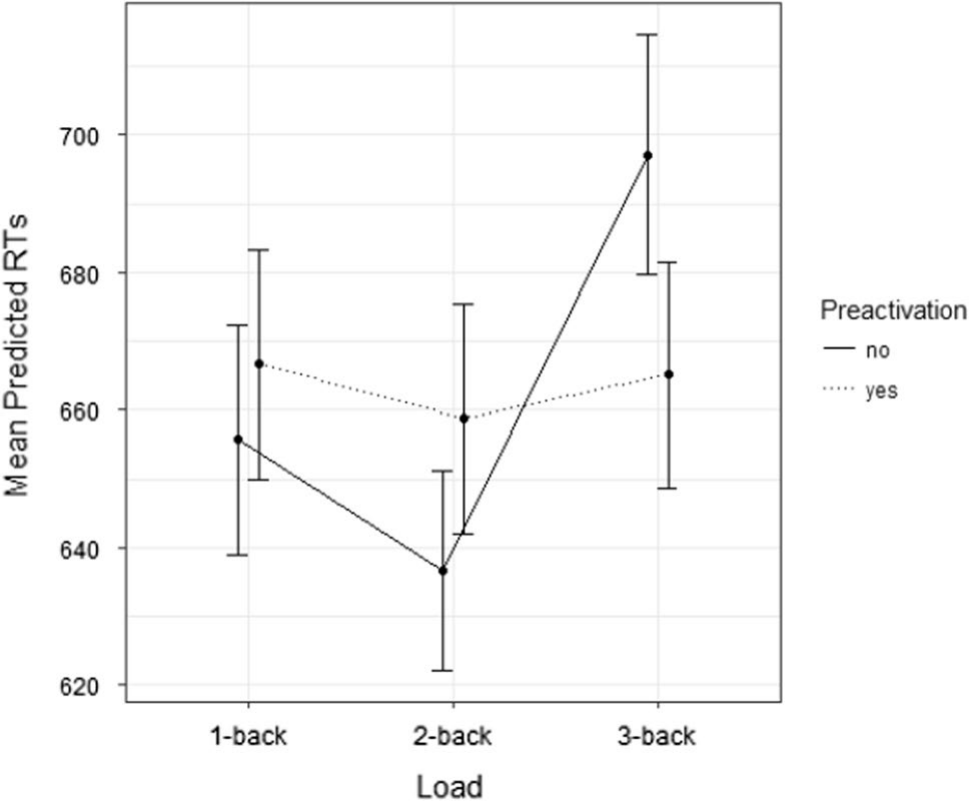
Experiment 2: mean predicted RTs (ms) as a function of semantic preactivation and load. Dots represent mean values, and the error bars represent 95% confidence interval

The model on participants’ RTs showed a significant Semantic preactivation * Load interaction (*F*(2, 610.88) = 4.030, *p* = 0.018) is shown in Fig. 4.

Multiple post-hoc comparisons based on preactivation showed that, only in the *3*-back condition, preactivated items (*M* = 673 ms) were identified faster than non-preactivated items (*M* = 706 ms; *t* = 2.303, *p* = 0.022). Comparisons based on load confirmed an effect only on non-preactivated items, since in *3*-back trials (*M* = 706 ms) targets were identified more slowly than *2*-back (*M* = 644 ms; *t* = 3.749*, p* = 0.001) and *1*-back trials (*M* = 663 ms; *t* = 3.002, *p* = 0.011). By contrast, preactivated items showed no differential effects of load (all *ts* < 0.3, all *ps* > 0.9).

## Discussion

Overall, we found preactivation benefits for RTs and especially in the more demanding load condition (i.e., *3*-back). We showed that, once preactivated, both taxonomic and thematic words are recognized faster than non-preactivated ones. Our results can be explained in the light of a generalized preactivation model, where target words are encoded prior to the *n*-back task and the words related to the target ones become more accessible and enhance target words encoding/recognition.

## General discussion

In the current study, we investigated how semantic relationship modulates WM updating process by using an *n*-back task ad hoc created. We found an advantage for semantically related words (vs. unrelated) regardless of their association type (i.e., taxonomic or thematic). Indeed, we found that semantic associated words were recognized more accurately and faster than those from unrelated associations. In addition, in Experiment 2, when we boosted semantic preactivation by bringing words into the broad focus of attention, findings also suggested that preactivation is effective and improves semantic superiority.

Semantically related words are better recognized than unrelated ones. The current finding of an advantage for updating strong semantic associations converges with Lendínez et al.’s (2014) findings. In fact, they showed facilitation effects during information updating. When the digits to update were more similar (i.e., in terms of numerical distance), substitution occurred faster. This is likely because the digit presented is closer to the digit stored in LTM; this overlap impacts similarity and ease of recall, and vice versa (see also Nairne 1990). Similarly to our findings, the closer the words were in the semantic node, the faster their recognition and update were. We could hypothesize that, when necessary to update a word that shares many features with another stored in memory (i.e., the same semantic script, node), the process can be performed more quickly; fewer features of the second word need activation because shared features are already activated.

The advantage for semantic relationship appears in contrast with findings on the detrimental impact of semantic relationship on recognition (e.g., Tse et al. 2011). However, as previously mentioned, these opposing findings could be based on task-demand artifacts. For example, where the words are more or less demanding in terms of explicit processing or a secondary task is presented, this is highly likely to elicit findings that diverge from ours.

A further important finding is the fact that we did not find differential processing in taxonomic and thematic relationship, as they both resulted in better, faster recognition than unrelated words. However, the absence of a specific semantic-organization effect is not entirely unexpected. As discussed above, the two semantic association types elicit different effects over the life span. In a developing cognitive system, there is differentiated use of semantic knowledge; for example, children first use thematic relations, then slot fillers, followed by taxonomic and hierarchical relations (i.e., logic/abstract categories) (see, e.g., Lucariello et al. 1992). This has been found not only at perceptual or implicit memory level, but also in explicit memory tasks such as those involving recall (e.g., Belacchi et al. 2011; Giofrè et al. 2017). For example, sensitivity detection for recognition of taxonomic associations was greater in older children (aged 9 to 10 years) than in younger ones (aged about 7 years), illustrating that taxonomies produce more interference in younger children. Presumably, younger children are not able to derive advantage from taxonomic associations, as older children do (Artuso et al. 2020). However, in adults, where the cognitive system is fully developed (e.g., university students recruited in the current study) taxonomic and thematic associated words are used more flexibly (Belacchi and Artuso 2018).

In Experiment 2, we emphasized semantic association through preactivation and thus boosted the effect generated by reading a word (i.e., with multiple semantic node activation). In addition, when participants were asked to produce words in a semantic context/node (i.e., preactivate related semantic associations) before doing the task, we observed (i) the efficacy of this pre-activation procedure and (ii) improvement of semantic superiority as observed in Experiment 1. However, this result is only observed in terms of recognition speed, as accuracy is at ceiling level (see Experiment 1 discussion). Indeed, activating (or preactivating) the semantic context of a given word is a mechanism supporting WM and updating and, thus, could potentially be a mechanism that favors word recognition in semantic associations (i.e., in contrast to those with no explicit associations).

In line with recent models of WM (i.e., as an emergent property of attention, perception and memory systems, e.g., Cowan 1999; Oberauer 2002), we can speculate that the preactivation process we devised is similar to bringing items into the broad focus of attention (via their preactivation in semantic LTM); these items are more carefully attended to, and are therefore more easily processed. In this instance, this was operationalized as more rapid recognition than non-preactivated items.

At this point, a methodological digression on updating tasks is also necessary. Updating was initially conceptualized as substitution of information (Morris and Jones 1990), and active process of binding/unbinding items (Artuso and Palladino 2011, 2018; Schmiedek et al. 2009). However, the *n*-back task, on the other hand, has substantially reduced task demands, and instead, is similar to continuous exposure to information flow. Although, arguably this makes the *n*-back a more ecological task, some doubts could be asserted as to whether this really measures updating per se. We know from the literature the *n*-back is also widely used in neuroimaging studies, especially for its ease of use in those contexts (e.g., Jonides et al. 1997). However, we believe it is important to note that some findings have illustrated the risks of using tasks with continuous presentation of stimuli; in this instance, this can ‘push’ participants toward adopting passive recency-based strategies (Palladino and Jarrold 2008).

It is worth limit our findings to the *n*-back task, a quite easy task based on low cognitive demands. We believe the absence of differences between taxonomic and thematic processing can be reasonably related to the low difficulty of the *n*-back. Indeed, when the task becomes more difficult, it is necessary to organize efficiently the information, to favor the economy of the cognitive system; therefore, in those instances, the use of taxonomies should be preferred, such as in dual tasks (e.g., Artuso et al. 2020; Belacchi and Palladino 2017). On the other hand, the absence of specific differences between taxonomic and thematic items may be a consequence of the general absence of differences in adults and their system flexibility (Belacchi and Artuso 2018).

Within our future aims we plan to design an experiment where thematic and taxonomic-related words are evaluated via the dimensions of valence, arousal and dominance, as suggested by Ishiguro and Saito (2021). This could give a more detailed picture of how semantic knowledge impacts WM. In addition, the innovation of this study will hopefully stimulate interest in devising updating tasks that take the semantic association between stimuli into consideration.

In sum, we have made an original contribution to understanding the role of semantic knowledge in WM function. In particular, we have demonstrated that semantic relationship is beneficial to the updating process in a *n*-back task. However, it is important to consider the level of the association (e.g., phonological, semantic), the stimuli (e.g., letter, word, digit), and the task (more active, i.e., requiring stimulus binding/unbinding of the stimuli, or less active, i.e., involving continuous presentation of stimuli, e.g., *n*-back task) to further investigate this topic.

## Open practice statement

All data are uploaded on Open Science Framework (link: https://osf.io/szykv/?view_only=f0a011d45af0466c81fe9b5bb96492c2) and will be made available upon request. None of the experiments was preregistered.[query].

## Appendix 1. Preliminary experiment on stimuli associative strength

A preliminary experiment was run to test the strength of the associative links between the three conditions we investigated (arbitrary, taxonomic and thematic relations). To compare the three conditions, we created a list of 180 word couples (60 arbitrary, 60 taxonomic and 60 thematic) by coupling the stimuli we used in the main experiments under different semantic relationships. All the possible couples were created (e.g., for the thematic yeast-flour-bread, participants were tested on the couples yeast-flour, yeast-bread, flour-bread; and so on, for all possible associations). The couples were then randomized into 3 different versions of the list (to control for order-related effects) and administered to 51 participants (*F* = 23; mean age = 35 ± 10.59), by asking them to evaluate the strength of the associative link in each couple on a 7-point Likert scale ranging from 1 (absolutely non-related) to 7 (absolutely related).

Their responses were analyzed in a mixed effects model that considered the associative score as dependent variable, the relationship type as 3-level fixed factor and random intercepts for participants and items. The results showed a statistically significant effect of the semantic relationship: *F*(2, 177) = 499.41, *p* < 0.001. When performing post-hoc comparisons with Tukey p-value correction for multiple comparisons, we observed that couples with arbitrary link (mean = 1.84) showed significantly lower scores compared to couples with taxonomic (mean = 5.68, *z* = − 27.554, *p* < 0.001) or thematic links (mean = 5.63, *z* = − 27.182, *p* < 0.001). However, the latter two categories did not show any significant differences (*z* = 0.052, *p* = 0.926).

## Appendix 2. Pilot experiment full set of stimuli

### Set A

#### Taxonomic triplets

**Table:**

Italiano	English
Negozio, farmacia, bar	Store, pharmacy, coffee shop
Mobile, sedia, letto	Furniture, chair, bed
Insetto, zanzara, mosca	Bug, mosquito, fly
Animale, cane, cigno	Animal, dog, swan
Stagione, primavera, autunno	Season, spring, fall
Colore, giallo, verde	Colour, yellow, green
Mobile, tavolo, armadio	Furniture, table, closet
Materia, Italiano, matematica	Subject, Italian, maths
Pianta, ortica, basilico	Plant, nettle, basil
Frutto, fragola, banana	Fruit, strawberry, banana
Persona, adulto, bambino	Individual, adult, child
Dolce, crostata, biscotto	Dessert, pie, cookie

#### Thematic triplets

**Table:**

Italiano	English
Luce, calore, fuoco	Light, heat, fire
Tetto, muro, casa	Roof, wall, house
Fusa, baffi, gatto	Purr, whiskers, cat
Bottiglia, specchio, vetro	Bottle, mirror, glass
Lievito, farina, pane	Yeast, flour, bread
Petalo, spina, rosa	Petal, thorn, rose
Criniera, ruggito, leone	Mane, roar, lion
Banco, lavagna, scuola	Desk, blackboard, school
Lavello, forno, cucina	Sink, oven, kitchen
Bicchiere, uva, vino	Cup, grapes, wine
Lama, manico, coltello	Blade, handle, knife
Manica, colletto, camicia	Sleeve, collar, shirt

### Set B

#### Taxonomic triplets

**Table:**

Italiano	English
Elemento, acqua, fuoco	Element, water, fire
Abitazione, castello, casa	Home, castle, house
Felino, tigre, gatto	Feline, tiger, cat
Materiale, legno, vetro	Material, wood, glass
Cibo, riso, pane	Food, rice, bread
Fiore, girasole, rosa	Flower, sunflower, rose
Animale, elefante, leone	Animal, elephant, lion
Istituto, banca, scuola	Institute, bank, school
Stanza, salotto, cucina	Room, living-room, kitchen
Bevanda, birra, vino	Beverage, beer, wine
Posata, forchetta, coltello	Cutlery, fork, knife
Indumento, pantaloni, camicia	Clothing, trousers, shirt

#### Thematic triplets

**Table:**

Italiano	English
Caffè, bibita, bar	Cafè, drink, coffee shop
Cuscino, coperta, letto	Pillow, blanket, bed
Stalla, ronzio, mosca	Stable, buz, fly
Becco, piume, cigno	Beak, plumage, swan
Vendemmia, settembre, autunno	Harvest, september, fall
Rana, stagno, verde	Frog, pond, green
Anta, ripiano, armadio	Shutter, shelf, closet
Numero, sottrazione, matematica	Number, subtraction, maths
Vaso, menta, basilico	Vase, mint, basil
Giallo, limone, banana	Yellow, lemon, banana
Palla, favola, bambino	Ball, fairy tale, child
Burro, farina, biscotto	Butter, flour, cookie

#### Arbitrary stimuli

**Table:**

Italiano	English
Regno	Kingdom
Sole	Sun
Costa	Shore
Pelle	Skin
Segno	Sign
Chiesa	Church
Corpo	Body
Pino	Pine
Gonna	Skirt
Cotone	Cotton
Vento	Wind
Aereo	Airplane

### Filler words

#### Italiano

Notte, nebbia, vaso, nuvola, carta, anello, foglia, insalata, veleno, fumo, freddo, panettone, madre, occhio, gruppo, punta, posto, capello, libro, città, festa, lettera, mamma, giorno, disegno, palazzo, pagina, arte, spalla, principe, porto, scala, filo, mondo, piede, guardia, sorella, sera, isola, albergo, collana, viaggio, banca, serpente, monte, punto, cielo, cuore, ponte, abito, sorriso, ragazza, bosco, luna, campo, moglie, pasta, faccia, soldato, lato, regione, forma, pietra, treno, numero, polizia, dente, erba, nuoto, dito, finestra, medico, pomodoro, lampada, scarpa, candela, tuffo, sapone, guanti, circo, coperta, uovo, fontana.

### English

Night, fog, vase, cloud, paper, ring, leaf, salad, poison, smoke, cold, panettone, mother, eye, group, tip, place, hair, book, city, party, letter, mom, day, drawing, building, page, art, shoulder, prince, harbour, staircase, wire, world, foot, guard, sister, evening, isle, hotel, necklace, trip, bank, snake, mountain, dot, sky, heart, bridge, suit, smile, girl, wood, moon, field, wife, pasta, face, soldier, side, region, shape, stone, train, number, police, tooth, grass, swimming, finger, window, physician, tomato, lamp, shoe, candle, dive, soap, gloves, circus, blanket, egg, fountain.

## Appendix 3. Mixed effects models specifications

### Experiment 1: Generalized mixed effect model on Accuracy

#### Random effects

**Table:**

Groups Name	Variance	SD	Corr.
Participant (Intercept)	0.6634	0.8145	

Number of obs: 900, groups: participants, 25

Please note: the model with random intercept on items could not converge

#### Fixed effects

**Table:**

Groups name	Estimate	SE	*z* Value
(Intercept)	3.47777	0.5573	6.240
Semantic relationship (arbitrary vs. thematic)	− 0.2411	0.6971	− 0.346
Semantic relationship (arbitrary vs. taxonomic)	− 0.4416	0.6728	− 0.656
Load (1-back vs. 2-back)	0.3055	0.7868	0.388
Load (1-back vs. 3-back)	− 1.5220	0.5953	− 2.557
Semantic relationship: load (arbitrary 1-back vs. thematic 2-back)	− 0.9656	0.9832	− 0.982
Semantic relationship: load (arbitrary 1-back vs. taxonomic 2-back)	− 0.8886	0.9603	− 0.925
Semantic relationship: load (arbitrary 1-back vs. thematic 3-back)	1.3216	0.8699	1.519
Semantic relationship: load (arbitrary 1-back vs. taxonomic 3-back)	1.9635	0.8986	2.185

### Experiment 1: Linear mixed effect model on log-transformed RTs

#### Random effects

**Table:**

Groups Name	Variance	SD	Corr.
Participant (Intercept)	0.01756	0.132529	
Item (Intercept)	0.00003	0.005602	
Residual	0.02767	0.166335	

Number of obs: 797, groups: item, 36; participants, 25

Please note: the model with random effects on neither participants nor items could converge

#### Fixed effects

**Table:**

Groups name	Estimate	SE	*t* Value
(Intercept)	6.55127	0.03186	205.608
Semantic relationship (arbitrary vs. thematic)	− 0.12015	0.02487	− 4.831
Semantic relationship (arbitrary vs. taxonomic)	− 0.07069	0.02501	− 2.826
Load (1-back vs. 2-back)	− 0.03090	0.02502	− 1.235
Load (1-back vs. 3-back)	− 0.03357	0.02621	− 1.281
Semantic relationship: load (arbitrary 1-back vs. thematic 2-back)	0.06392	0.03553	1.799
Semantic relationship: load (arbitrary 1-back vs. taxonomic 2-back)	0.03161	0.03556	0.889
Semantic relationship: load (arbitrary 1-back vs. thematic 3-back)	0.11317	0.03623	3.124
Semantic relationship: load (arbitrary 1-back vs. taxonomic 3-back)	0.02071	0.03610	0.574

### Experiment 2: Generalized mixed effect model on accuracy

#### Random effects

**Table:**

Groups name	Variance	SD	Corr.
Participant (Intercept)	1.063	1.031	

Number of obs: 720, groups: participants, 30

Please note: the model with random intercept on items could not converge

#### Fixed effects

**Table:**

Groups name	Estimate	SE	*z* Value
(Intercept)	6.31895	3.06227	2.063
Preactivation (no vs. yes)	0.01541	1.45413	0.011
Semantic relationship (thematic vs. taxonomic)	− 1.19373	1.19718	− 0.997
Load (1-back vs. 2-back)	− 1.18832	1.19777	− 0.992
Load (1-back vs. 3-back)	− 1.79647	1.13942	− 1.577
Vocabulary	− 2.06167	3.49901	− 0.589
Preactivation: load (no 1-back vs. yes 2-back)	0.42624	1.74532	0.244
Preactivation: load (no 1-back vs. yes 3-back)	− 0.01568	1.61327	− 0.010
Preactivation: semantic rel. (no thematic vs. yes taxonomic)	0.42878	1.74459	0.246
Semantic relationship: load (thematic 1-back vs. taxonomic 2-back)	0.84960	1.45279	0.585
Semantic relationship: LOAD (thematic 1-back vs. taxonomic 3-back)	1.19272	1.38610	0.860
Preactivation: semantic relationship: load (no thematic 1-back vs. yes taxonomic 2-back)	− 1.55378	2.11163	− 0.736
Preactivation: semantic relationship: load (no thematic 1-back vs. yes taxonomic 3-back)	0.63041	2.08039	0.303

### Experiment 2: Linear mixed effect model on log-transformed RTs

#### Random effects

**Table:**

Groups name	Variance	SD	Corr.
Participant (intercept)	0.0182978	0.13527	
Item (intercept)	0.0002622	0.01619	
Residual	0.0236295	0.15372	

Number of obs: 655, groups: item, 24; participants, 30

Please note: the model with random effects on neither participants nor items could converge

#### Fixed effects

**Table:**

Groups name	Estimate	SE	*t* Value
(Intercept)	6.18192	0.27201	22.72712
Preactivation (no vs. yes)	0.01635	0.02897	0.56435
Semantic relationship (thematic vs. taxonomic)	0.04136	0.03144	1.31568
Load (1-back vs. 2-back)	− 0.02642	0.03107	− 0.85031
Load (1-back vs. 3-back)	0.11576	0.03158	3.66610
Vocabulary	0.34122	0.33094	1.03105
Preactivation: load (no 1-back vs. yes 2-back)	0.01207	0.04103	0.29426
Preactivation: load (no 1-back vs. yes 3-back)	− 0.11222	0.04169	− 2.69177
Preactivation: semantic rel. (no thematic vs. yes taxonomic)	0.00002	0.04162	0.00057
Semantic relationship: load (thematic 1-back vs. taxonomic 2-back)	0.01940	0.04446	0.43626
Semantic relationship: load (thematic 1-back vs. taxonomic 3-back)	− 0.09708	0.04471	− 2.17141
Preactivation: semantic relationship: load (no thematic 1-back vs. yes taxonomic 2-back)	0.00415	0.05912	0.07017
Preactivation: semantic relationship: load (no thematic 1-back vs. yes taxonomic 3-back)	0.09489	0.05904	1.60726

## Footnotes

1 We obtained a ceiling effect for accuracy in both Experiments. We believe it is pertinent here to raise the issue concerning measurement of updating. Traditionally, updating tasks (such as the running memory span task and other tasks derived from it) have measured updating indirectly, through the index of recall accuracy. However, recall accuracy tends to combine the effects of all processes that are active during updating and to mask their separate contributions (e.g., Artuso & Palladino, 2014; Palladino & Jarrold, 2008). We have pointed out that recall accuracy reflects non-specific memory success (accuracy), and for this reason, a less ambiguous measurement of the process, such as RTs, would be useful (e.g., Artuso & Palladino, 2011). Indeed, in different papers we used tasks to obtain ceiling accuracy and thus focus on RTs as the crucial measure (e.g., Artuso & Palladino, 2011, 2014, 2018). This was the case also with the current n-back task that usually represents a task with ceiling accuracy and where RTs are more informative.
